# Occupational Asthma in Antibiotic Manufacturing Workers: Case Reports and Systematic Review

**DOI:** 10.1155/2011/365683

**Published:** 2011-03-17

**Authors:** Sara Díaz Angulo, Joanna Szram, Jenny Welch, Julie Cannon, Paul Cullinan

**Affiliations:** ^1^Centro Medico de Asturias, Avendia José María Richard, Oviedo, 33193 Asturias, Spain; ^2^Department of Occupational & Environmental Medicine, National Heart & Lung Institute (Imperial College) and Royal Brompton Hospital, Sydney Street, London SW3 6NP, UK

## Abstract

*Background*. The risks of occupational asthma (OA) from antibiotics are uncertain. We report 4 new cases and a systematic review of the literature. *Methods*. Cases were identified through a specialist clinic, each underwent specific provocation testing (SPT). We subsequently reviewed the published literature. *Results*. The patients were employed in the manufacture of antibiotics; penicillins were implicated in three cases, in the fourth erythromycin, not previously reported to cause OA. In two, there was evidence of specific IgE sensitisation. At SPT each developed a late asthmatic reaction and increased bronchial hyperresponsiveness. 36 case reports have been previously published, 26 (citing penicillins or cephalosporins). Seven cross-sectional workplace-based surveys found prevalences of 5–8%. *Conclusions*. OA in antibiotic manufacturers may be more common than is generally recognised. Its pathogenesis remains unclear; immunological tests are of uncertain value and potential cases require confirmation with SPT. Further study of its frequency, mechanisms, and diagnosis is required.

## 1. Introduction

Among the several hundred workplace agents implicated as causes of occupational asthma (OA), several are encountered in the pharmaceutical manufacturing industry [1]. These include antibiotics which, through inhalation, may induce asthma in exposed employees. The role of dermal exposure and the detailed pathogenesis of the condition—as with many other low molecular mass causes of OA—remain unclear. Immunological tests are of uncertain value and most diagnoses require confirmation with specific provocation testing.

The first case of occupational antibiotic allergy was described in 1953, but a relatively small number of individual case reports have been published subsequently. Very large quantities of antibiotics are produced in almost every country but the frequency of OA in those who manufacture them remains unclear. While prevalence estimates of around 10% have been reported [2–4], clinical experience suggests a far less common problem. Here we report three new cases of occupationally-induced penicillin allergy and, for the first time, a case of asthma induced by work in erythromycin manufacture. The reports are supplemented by a systematic literature review of antibiotic occupational asthma.

## 2. Methods

### 2.1. Case Reports

New cases were seen and diagnosed at Royal Brompton Hospital in London, UK between 1995 and 2009. Specific IgE measurements, where attempted, were carried out by either the radioallergosorbent test (RAST) method or the commercial ImmunoCAP assay. Each patient underwent controlled, single-blind, specific provocation testing using a dust-tipping method, with small quantities of the relevant antibiotic powder mixed with a larger amount of dried lactose; active and control exposures were carried out on sequential days with the patients having stopped any asthma medications prior to the tests. Responses were assessed using serial FEV1 measurements following challenge and by changes in bronchial responsiveness to inhaled histamine using the Yan technique [5].

### 2.2. Systematic Review of Literature

We were not able to identify any previous systematic review of this subject. We searched the published literature using the Medline database between 1953 and February 2010. Both key word- and text word-based searches were performed with combinations of the terms “occupational asthma”, “asthma”, “respiratory sensitisation”, and “antibiotics”. In addition, we examined the reference lists of relevant articles. Both case reports (*n* = 21 papers) and workforce studies (*n* = 7) were retrieved (one case report in Russian was not included.) and their information extracted on to a standard form independently by two reviewers.

We used a quantitative structure-activity relationship (qSAR) model to examine the potential for each published antibiotic to act as a respiratory sensitising agent. “Hazard indices” for each were calculated, where possible, using the Chemical Asthma Hazard Assessment Program [6] which generates a risk prediction for organic compounds with a molecular mass of less than 1 kDa.

## 3. Findings

### 3.1. Case Reports (Table 1, Figure 1)

Each of the four cases was involved in the primary manufacture or formulation of antibiotics in the United Kingdom and had presented with new onset, work-related asthmatic symptoms. Three worked with penicillins and their derivatives, the fourth with a variety of medicines including erythromycin. This last, a woman of 52, developed asthma 22 years previously, two years after starting work on the packaging lines. She reported that her symptoms worsened with exposure to granulated erythromycin and on two occasions in the year prior to referral had required treatment with oral corticosteroids. At referral, she had normal spirometry, was demonstrated to be atopic on skin prick testing, and completed a series of peak flow measurements which showed significant variability on both work and rest days, with no clear work-related pattern.

**Figure 1:**
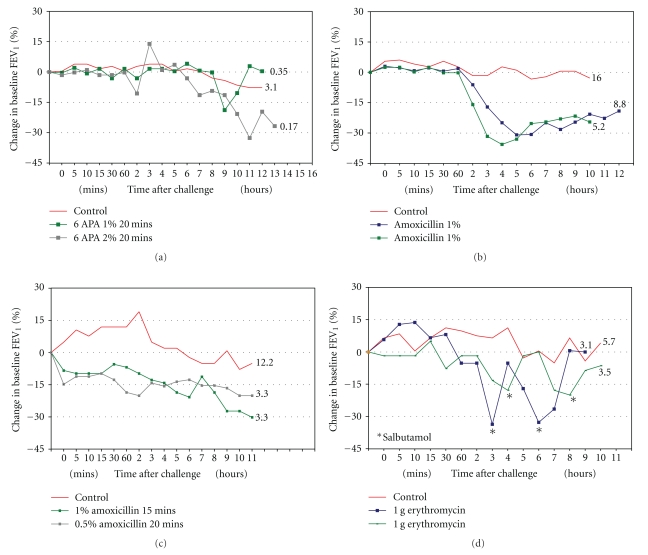
Changes in FEV_1_ and histamine reactivity following bronchial provocation testing in four antibiotic manufacturing workers. Serial FEV_1_ measurements (*y*-axis) are plotted against time after provocation (*x*-axis); a fall of greater than 15% from baseline indicated a significant response. A late reaction (greater than two hours after challenge) is seen in all cases; an early reaction was also seen in case c. The numerical value associated with each challenge plot is the postchallenge histamine PC_20_ result (mg/ml histamine): the lower the PC_20_ the greater the degree of bronchial hyperresponsiveness.

All four patients underwent single-blind, controlled specific provocation testing using a dust-tipping method. Each of the four developed a (predominantly) late asthmatic reaction (Figure 1) accompanied by increased bronchial hyperresponsiveness as demonstrated by a fall in histamine PC_20_ concentration following challenge; these findings were repeatable on further testing but not seen after identical exposure to lactose powder alone. Following diagnosis, each patient avoided further exposure at work to the causative antibiotic with improvement or resolution of their symptoms. They were advised to avoid therapeutic use of the relevant antibiotic.

### 3.2. Literature Review (Table 2)

Previous published case reports, including a total of 37 patients, are summarised in Table 2. All but one of the cases was employed in the manufacture of antibiotics. In 25 individuals, the diagnosis was supported by specific inhalation testing which prompted early, late, and dual asthmatic responses in 14 (56%), six (24%), and three (12%) patients, respectively, with the results of the remaining two positive tests being unspecified. A hazard index was calculated for all bar two of the implicated antibiotics (the exceptions being vancomycin and colomycin, both of which have a molecular mass greater than 1 kDa) and was in all cases between 0.99 and 1.00.

Three reports describe patients sensitised to penicillins. In the first, published in 1953, Eaton Roberts described two employees in a US factory with clinical evidence of OA ascribed to penicillin; apart from skin prick testing with procaine penicillin (negative), he did not perform any objective investigations [7]. Similarly, Tara in 1957 [8] and Gaultier et al. in 1960 [9] reported a total of six French workers with clinical evidence of new-onset asthma after exposure to penicillin dust.

Synthetic penicillins have also been reported as a cause of work-related asthma. Davies et al. [10] used specific provocation testing to confirm OA from inhaled ampicillin in three UK employees; in each case skin prick tests with ampicillin and a variety of penicillin antigens were negative. Oral challenge with ampicillin induced asthmatic and other allergic symptoms in two of the three cases. Losada et al. described two cases of dyspnoea after inhalation of semisynthetic penicillins in workers in the manufacture of antibiotics [11]; in neither case was there objective evidence of immune sensitisation to these antibiotics. In Germany, Wuthrich and Hartmann [12] described a single case of OA from ampicillin; specific IgE antibodies to benzylpenicilloyl were detected and cumulative inhalation testing with lactose, tetracycline, ampicillin, and chloramphenicol caused a delayed asthmatic reaction. A factory worker in Belgium developed OA from amoxicillin [13] and subsequently, while employed as a nurse, to latex. Piperacillin, a semisynthetic penicillin, has also been reported to cause OA, the diagnosis supported by both skin prick testing and an immediate asthmatic response to specific inhalational testing [26].

There are several reports of cephalosporins and associated precursors and derivatives as causative agents for OA; 11 cases are summarised in Table 2 with supporting evidence from specific provocation testing in ten, albeit most frequently reported as immediate asthmatic responses only. Serum and skin prick tests of immunological sensitisation were only occasionally positive [15, 19, 20], and oral challenges, both positive, were reported in just two patients [11, 18].

The remaining eight reported cases developed OA during the manufacture of antibiotics other than penicillins or cephalosporins. Most have been in workers exposed to the macrolide spiramycin, an antibiotic widely used in the livestock industry and particularly with poultry so that traces may be found in some chicken eggs. Davies and Pepys [23] described the case of a pharmaceutical worker in whom the diagnosis of OA from spiramycin was confirmed by specific inhalation challenge; he reported symptoms on eating eggs but had negative skin prick tests to egg extracts. Six other cases, in Canadian and Italian manufacturers [4, 25], have been reported, in each case following specific inhalation testing. In one case [25], an immediate asthmatic response to provocation testing was also elicited by adipic acid, an additive used to bind spiramycin. In the only case of reported antibiotic OA outside the manufacturing sector, Paggiaro et al. [24] described dermatitis and asthma due to spiramycin in a chick breeder; a skin prick test to spiramycin was positive as was specific inhalation testing with chick feed containing the antibiotic.

Menon and Das [22] documented in an Indian worker immediate asthmatic reactions to intradermal, inhalation and oral testing with tetracycline but no response to inhalation testing with two antibiotics (nystatin and chloromycetin) to which he was not exposed at work. Three cases of OA from thiamphenicol—a methyl-sulfonyl analogue of chloramphenicol—were reported by Ye et al. [27]. Vancomycin has been described as a cause of OA in a single patient [28], the diagnosis confirmed by serial peak flow measurements. The most recently reported case was of OA due to the polymyxin antibiotic colomycin in an antibiotic transport and storage worker, who demonstrated an early asthmatic reaction following specific inhalation challenge [29]; no evidence of specific IgE was found despite extensive in vitro immunological testing.

### 3.3. Workforce Studies

We identified seven reports of epidemiological studies carried out in antibiotic manufacturing sites; all but one was of cross-sectional design. In several cases, the absence of detailed information on the size of the exposed populations precludes any estimate of disease prevalence.

Briatico-Vangosta et al. [2] surveyed 91 Italian workers exposed to cephalosporins with a combination of a symptoms questionnaire, skin prick and intradermal testing, and “on-off” testing of asthma symptoms and pulmonary function. On this basis, OA was reported for seven (8%) employees. Skin testing produced immediate-type responses in five workers with OA (in three cases with prick testing) suggesting to the authors that the asthma had, in some cases at least, arisen through a specific IgE-related mechanism.

Chida and Uehata [30] surveyed by interview 24 employees of a pharmaceutical factory which produced two antibiotics (ampicillin and cephalexin) as well as an antispasmodic and three anti-inflammatory drugs. Those with respiratory symptoms (*n* = 18) underwent skin prick, serum, and pulmonary function testing. Probable antibiotic-related asthma was claimed for four employees, two of whom had immediate responses to skin testing with ampicillin. In the absence of any clear information on the population at risk, a prevalence estimate is not available.

Phenylglycine acid chloride (PG-AC) is a highly reactive compound used chiefly in the manufacture of ampicillin and other antibiotic side chains. A survey of 24 workers involved in the production of PG-AC [3] included a symptoms questionnaire, examination of occupational health records, skin testing, and spirometry. Seven workers were felt, on the basis of their history alone, to have a respiratory allergy to PG-AC; all had positive responses to prick and/or intradermal skin testing with the conjugated compound. Two were admitted to hospital for specific inhalation testing with positive findings. A further seven employees were deemed to have “irritant” respiratory responses to PG-AC (all skin tests negative) and in nine the history was considered “equivocal”; two of this last group had positive skin test results. The authors commented that the amino groups in PG-AC predispose to the formation of hapten-protein conjugates and hence its allergenicity. Again, in the absence of any information on the size of the exposed workforce, an estimate of prevalence in this setting is unavailable.

Carnevale et al. [31] studied 67 workers employed in manufacturing and encapsulating antibiotics in Italy. Following questionnaire, clinical and laboratory investigations, two (3%) cases of OA due to ampicillin were recorded. In both cases skin tests were negative. Post- and preshift urine testing confirmed the systemic absorption of ampicillin by these workers. A similar survey was reported by Carlesi et al. [32]. Among 26 employees in an antibiotic manufacturing plant, eight (31%) claimed respiratory symptoms which in two cases (8%) were suggestive of asthma. A further three employees had positive skin prick tests to penicillin G and/or amoxicillin.

Two surveys of workers involved in spiramycin manufacture have been reported. Malo and Cartier [4] investigated all 51 employees at a processing plant in Canada. Twelve, on the basis of a compatible history or evidence of (work-related) bronchial hyperreactivity, underwent specific inhalation testing with positive findings in four cases. The measured prevalence of 8% was considered by the authors, to be a minimum estimate. In Italy, a 12-year prospective study of 305 workers in a spiramycin-manufacturing plant [33] suggested work-related asthma in 15 (5%), all of whom had positive epicutaneous or intradermal skin tests to spiramycin. Four had additional symptoms of rhinitis and one of urticaria. In the full study population, 41 employees (13%) had positive skin tests, 37 (90%) of them with allergic symptoms of some kind.

## 4. Discussion

Our cases add three to the previously reported eight cases of OA attributed to inhalation of synthetic penicillins during their manufacture, and the first case of disease arising from workplace exposure to erythromycin. An additional seven cases of penicillin OA were reported to a UK national surveillance scheme between 1989 and 2009 (THOR personal communication).

An examination of published findings from surveillance schemes in other parts of the world found specific reference to antibiotics in none although the Propulse scheme in Quebec collated seven cases of OA (2.4% of the total) attributed to “medical drugs” [34]. Our systematic review of the remaining literature revealed a total of 37 cases published over a period of almost 50 years, although others were identified during the course of seven epidemiological studies, the most recent of which was published over 20 years ago. It was often difficult, in reviewing the workplace surveys, to obtain meaningful estimates of prevalence but what information was available suggests that about 10% of those surveyed had disease suggestive of OA. Thus since the first report in 1953 there appears to have been only sporadic attention paid to what may be a significant occupational risk.

The clinical features of the published cases are similar to those found in other examples of allergic OA and are broadly indicative of an immediate-type respiratory hypersensitivity. A latent period of asymptomatic exposure was reported for all, usually of fewer than 24 months although occasionally far longer, and rhinitis was a frequent accompaniment to asthma symptoms. In those cases where specific inhalation testing was used in diagnosis and the findings reported in full, a late or dual asthmatic response was reported in 43%. In most cases of isolated early asthmatic responses to specific provocation, there was evidence of immunological sensitisation on skin or serum testing. Each of the implicated antibiotics had a very high hazard index derived from a quantitative structure-activity relationship analysis [35]; in the clinical context of a patient with characteristic symptoms, index values such as these have a high positive predictive value for OA [36]. Finally, Roberts [7] reported the apparently successful, subcutaneous desensitisation of two patients with OA from penicillin.

Nonetheless, as with many other low-molecular-weight causes of the disease, the immunological details of antibiotic-related OA remain unclear. While an IgE-associated mechanism is likely in many cases, the possibility of sensitisation arising from an alternative mechanism, perhaps through cross-linking of cell surface receptors, cannot be ruled out. Even when, as is not always the case, their techniques are described, the variety of skin test methods used in the published reports makes their interpretation and diagnostic significance uncertain. In any case, the results were often negative and where they were not there is no systematic information on the findings among exposed but nonasthmatic employees. Similar comments apply to the use of tests for serum-specific IgE antibodies which were less often performed and more often negative although some success has been recorded for in-house assays using cephalosporin conjugates [20, 21] or thiamphenicol [27]. Thus, and probably in contrast to their use in oral antibiotic allergy [37], the value of available tests for evidence of immunological sensitisation in this context remains unclear.

Penicillins and cephalosporins are common causes of oral drug allergy which in most cases is attributed to an IgE-associated immune response to one or more hapten-protein conjugates. Penicillins are composed of betalactam and thiazolidine rings with one or more differentiating side chains; the instability of the betalactam causes its carbonyl group to form amide-bonds with the amino groups of lysine residues from nearby proteins. “Minor” antigenic determinants, some formed from side chain-protein conjugates, may also induce IgE immune responses. 7 aminocephalosporanic acid, the active nucleus of cephalosporins, is structurally similar to the active nucleus of the penicillins, consisting of betalactam and 6 dyhydrothiazolidine rings. Hapten-protein conjugates of the cephalosporin betalactam are relatively unstable. IgE antibodies that react to cephalosporins detect a large number of specific antigens derived from protein conjugates formed from the side chain(s), the side chain plus a portion of the betalactam ring, or the complete cephalosporin. On the basis of ELISA inhibition assays in two patients with OA from cefteram, Suh et al. [19] suggested that specific IgE responses to the cephalosporin may involve haptens, new antigenic determinants, or both and are likely to vary between patients.

Seven of the identified case reports describe oral challenges to the causative antibiotic (in six cases a penicillin or cephalosporin) in patients with OA. All but one produced an allergic response. The three cases reported by Davies et al. [10] had subtly different responses to inhaled and oral (negative in one patient) provocation tests suggesting the possibility that individually they were responsive to different hapten-protein antigens. The patient described by Jimenez et al. [14] had allergic responses to both inhaled and oral amoxicillin, but not to oral penicillin V suggesting to the authors that the responsible antigenic determinant lay in the aminohydroxyphenyl side-chain of amoxicillin. In contrast, Sastre et al. [18] reported the case of a patient with OA attributed to cefadroxil, a cephalosporin that shares a side chain with amoxicillin. Oral challenge with cefadroxil, but not with amoxicillin, produced an asthmatic response suggesting that her sensitisation was to the dihydrothiazine cephalosporin ring rather than to its side chain. Losada et al. [11] reported a further patient with asthmatic responses to both inhaled and oral cephalosporin but not to penicillin G. Oral allergy may also develop in patients with OA attributed to other antibiotics [22]. While the evidence base is weak, and we cannot exclude the possibility of bias in those selected for oral challenge or in publication, we suggest that there is sufficient experience to advise that patients with OA from an inhaled antibiotic encountered at work should avoid taking the same—or closely related—drug by mouth unless its safety has been established by a carefully supervised provocation test.

We have been unable to find valid information on the numbers of exposed employees in any parts of the world but we expect these to number many thousands. Much manufacture is carried out in conditions where exposures to employees are very low but this is not always the case, and we note the general movement of pharmaceutical manufacturing away from its traditional base in western Europe and North America. We note also that most published evidence relates to older types of antibiotic. This is likely to reflect the number and exposures of those involved in their manufacture rather than any intrinsic hazard; the hazard indices for newer antibiotics such as flucloxacillin (0.909), clarithromycin (0.997), tobramycin (1.0), and azithromycin (1.0) are no lower. While cases of OA arising from antibiotic manufacture are rarely published, the available epidemiological evidence, admittedly scanty, sometimes deficient and all of it dated, suggests that the risks may be higher than many appreciate. We suggest that further workplace-based surveys, of careful design and supported by improvements in immunological diagnosis, are required.

## Figures and Tables

**Table 1:** Cases of occupational asthma from antibiotics identified at Royal Brompton Hospital in the period 1995–2009.

case	Year of diagnosis	Workplace exposure	Allergic symptoms	Latency	Specific IgE	Bronchial provocation test		
						Agent	FEV1 response	Increase in histamine reactivity
a	1995	penicillin	wheeze	19 years	not done	penicillin	late	Yes
b	1996	amoxicillin	wheeze, cough	27 years	penicilloyl G (+) penicilloyl V (+)	amoxicillin	late	yes
c	2000	amoxicillin	wheeze, cough	27 years	amoxicilloyl (+)	amoxicillin	late	yes
d	2009	erythromycin	wheeze, rhinitis	2 years	erythromycin ethylsuccinate (−)	erythromycin ethylsuccinate	late	Yes

**Table 2:** Published cases of occupational allergy to antibiotics 1953–2009.

Penicillins										
Reference	Year	Country	No.	Exposure	Latency	Respiratory symptoms	Skin test	Specific IgE	Bronchial provocation test	Oral challenge

[7]	1953	USA	2	penicillin	#1: “weeks” #2: 1 year	#1: cough, rhinitis #2: cough, rhinitis, wheeze	Procaine penicillin (+)	not reported	not reported	not reported
[8]	1957	France	4	penicillin	#1: “years” #2: 3 months #3: 1 year #4: 1 year	#1: cough, rhinitis, dyspnoea #2: dyspnoea, eczema #3: dyspnoea #4: asthma, dyspnoea, eczema	not reported	not reported	not reported	not reported
[9]	1960	France	2	penicillin	#1: 1 year #2: 7 years	#1: asthma, urticaria #2: asthma	#1: PMP* (+) #2: penicillin (+)	not reported	not reported	not reported
[10]	1974	UK	3	ampicillin BP* 6 APA*	#1: 2 years #2: 2 years #3: 2 years	asthma (*n* = 3) rhinitis (*n* = 2) eczema (*n* = 2) conjunctivitis (*n* = 1)	ampicillin (−) ampicillin polymer (−) BPP* (−) MDM* (−)	not reported	#1: ampicillin (+LR*)6APA* (+/−) #2: ampicillin (+LR*) BP*(+LR*) commercial 6APA* (+LR*) purified 6APA* (−) BPP* (−) #3: ampicillin (+LR*) BP* (+LR*) 6APA* (−)	#1: ampicillin (−) #2: ampicillin (+LR*) and intestinal symptoms #3: BP* (+ER*) and urticaria
[11]	1980	Spain	2	amoxicillin ampicillin	#1: 1 year #2: 1 year	#1: rhinitis, dyspnoea, wheeze #2: cough, wheeze	not reported	negative	not reported	not reported
[12]	1982	Germany	1	ampicillin	NS*	cough, rhinitis, dyspnoea, fever	ampicillin (−)	BPP* (+)	antibiotic mix (+LR*)	not reported
[13] ne	1997	Belgium	1	amoxicillin	6 months	cough, wheeze, rhinitis	not reported	not reported	amoxicillin (+ER* LR*)	not reported
[14]	1998	Spain	1	amoxicillin	27 years	cough, rhinitis, wheeze, dyspnoea	amoxicillin (−) ampicillin (−) BP* (−) BPP* (−) MDM* (−)	amoxicillin (+) ampicillin (−) (penicillin V (−)	amoxicillin: (+ER*) penicillin V (−)	amoxicillin (+LR*) penicillin V (−)

Cephalosporins										

Reference	Year	Country	No.	Exposure	Latency	Allergic symptoms	Skin test	Specific IgE	Bronchial provocation test	Oral challenge

[11]	1980	Spain	1	cephalexin,	3 months	cough, wheeze	PP* (−) penicillin G (−)	negative	not reported	cephalosporin (NS) (+) and rhinitis, urticaria
[15]	1981	UK	2	7ACA* 7CTD*	#1: NS* #2: 10 years	#1: cough, rhinitis, chest tightness #2: chest tightness, dyspnoea	#1: 7ACA (+) 7CTD (+) cefalexin (−) #2: cephalexin (+)	not reported	#1: 7ACA (+ER*) 7CTD (+ER*) cephalexin (−) #2: cephalexin (+ER*)	not reported
[16]	1995	UK	1	ceftazidime	1 year	rhinitis, dyspnoea	not reported	not reported	ceftazidime (+ER* LR*)	not reported
[17]	1996	Italy	1	cefmetazole 7-ACA*	1 year	cough, rhinitis, bronchospasm	cefmetazole (−) 7-ACA* (−)	penicillin G (−) penicillin V (−)	cefmetazole (+NS*) 7-ACA (+NS*)	not reported
[18]	1999	Spain	1	cefadroxil	9 months	cough, rhinitis, dyspnoea, chest tightness	PP* (−) MDM* (−) BP* (−) amoxicillin (−) cefadroxil (−)	penicillin G (−) penicillin V (−) amoxicillin (−) ampicillin (−) cefaclor (−)	cefadroxil (+ER*)	amoxicillin (−) cephalexin (+ER*)
[19]	2003	Korea	2	cefteram	NS*	NS*	#1: cefteram (+) #2: cefteram (+)	#1: cefteram-HSA* (+) #2: cefteram-HSA* (+)	#1: cefteram (+ER*) #2: cefteram (+ER*)	not reported
[20]	2004	Korea	2	7-ACA* ceftriaxone	2 years	#1: rhinitis, respiratory symptoms #2: rhinitis, respiratory symptoms	#1: 7-ACA* (+) ceftriaxone (+) #2: 7-ACA* (−) ceftriaxone (−)	#1: 7-ACA-HSA* (+) #2: 7-ACA-HAS* (−)	#1: 7-ACA* (+ER*) ceftriaxone (−) #2: 7-ACA* (+NS*) ceftriaxone (−)	not reported
[21]	2009	Italy	1	7-TACA* Cephalosporins	8 months	cough, rhinitis, dyspnoea	not reported	not reported	7-TACA*: (+ER*)	not reported

Miscellaneous										

Reference	Year	Country	No.	Exposure	Latency	Allergic symptoms	Skin test	Specific IgE	Bronchial provocation test	Oral challenge

[22]	1977	India	1	tetracycline	1 year	cough, wheeze, dyspnoea	not reported	not reported	tetracycline (+ER*)	tetracycline (+ER*) and urticaria
[23]	1975	UK	1	spiramycin	1 year	cough, rhinitis, dyspnoea, dermatitis	spiramycin (+)	not reported	spiramycin (+LR*)	not reported
[24]	1979	Italy	1	spiramycin	1 year	cough, asthma, dermatitis	spiramycin (+)	not reported	chick feed with spiramycin (+LR*)	not reported
[25]	1984	Italy	2	spiramycin adipid acid	#1:14 years #2:7 months	#1: dyspnoea #2: cough, dyspnoea	not reported	not reported	#1: spiramycin adipate (+ER* LR*) #2: spiramycin adipate (+ER*) spiramycin base (+ER* LR*)	not reported
[26]	1995	Italy	1	piperacillin	22 months	rhinitis, dyspnoea, wheeze, rash	piperacillin (+)	not reported	piperacillin (+ER*)	not reported
[27]	2006	Korea	2	thiamphenicol	NS*	#1: rhinitis, asthma #2: rhinitis, asthma	#1: thiamphenicol (+) #2: thiamphenicol (+)	#1: thiamphenicol (+) #2 thiamphenicol (+)	#1: thiamphenicol (+ER*) #2: thiamphenicol (+ER*)	not reported
[28]	2009	Korea	1	vancomycin	5 months	rhinitis, chest tightness	vancomycin (−)	vancomycin-HSA* (−)	not reported	not reported
[29]	2010	Spain	1	colomycin	3 months	rhinitis, cough, wheeze, dyspnoea	not reported	negative	colomycin (+ER*)	not reported

*NS: not specified, *ER: early (asthmatic) response, *LR: late (asthmatic) response.

*HSA: human serum albumin, *MDM: minor determinant (penicillin) mix, *BP: benzylpenicillin.

*(B)PP: (benzyl)penicilloyl polylysine, *PMP: phenoxymethyl penicillin, *6APA: 6 amino penicillanic acid, *7-ACA: 7aminocephalosporanic acid, *7CTD: tosylate dihydrate derivative of 7ACA.

*7-TACA: 7-amino-3thiometihyl-3-cephalosporanic acid.
